# The Effect of Coordinating the Outpatient Treatment across Medical Specialities for Patients With Multimorbidity

**DOI:** 10.5334/ijic.7535

**Published:** 2024-04-09

**Authors:** Cathrine Bell, Charlotte Weiling Appel, Anders Prior, Anne Frølich, Asger Roer Pedersen, Peter Vedsted

**Affiliations:** 1 Medical Diagnostic Centre, University Research Clinic for Innovative Patient Pathways, Regional Hospital Central Jutland, Department of Clinical Medicine, Central Denmark Region, Denmark; 2 Research Unit for General Practice, Aarhus, Denmark; 3 Innovation and Research Centre for Multimorbidity, Slagelse Hospital, Region Zealand, Denmark; 4 Centre for General Practice, Faculty of Health and Medical Sciences, University of Copenhagen, Denmark

**Keywords:** outpatients, hospitals, multimorbidity, delivery of health care, intervention, Denmark

## Abstract

**Introduction:**

Patients with multimorbidity attend multiple outpatient clinics. We assessed the effects on hospital use of scheduling several outpatient appointments to same-day visits in a multidisciplinary outpatient pathway (MOP).

**Methods:**

This study used a quasi-experimental design. Eligible patients had multimorbidity, were aged ≥18 years and attended ≥2 outpatient clinics in five different specialties. Patients were identified through forthcoming appointments from August 2018 to March 2020 and divided into intervention group (alignment of appointments) and comparison group (no alignment). We used patient questionnaires and paired analyses to study care integration and treatment burden. Using negative binomial regression, we estimated healthcare utilisation as incidence rates ratios (IRRs) at one year before and one year after baseline for both groups and compared IRR ratios (IRRRs).

**Results:**

Intervention patients had a 19% reduction in hospital visits (IRRR: 0.81, 95% CI: 0.70–0.96) and a 17% reduction in blood samples (IRRR: 0.83, 0.73–0.96) compared to comparison patients. No effects were found for care integration, treatment burden, outpatient contacts, terminated outpatient trajectories, hospital admissions, days of admission or GP contacts.

**Conclusion:**

The MOP seemed to reduce the number of hospital visits and blood samples. These results should be further investigated in studies exploring the coordination of outpatient care for multimorbidity.

**Research question:**

Can an intervention of coordinating outpatient appointments to same-day visits combined with a multidisciplinary conference influence the utilisation of healthcare services and the patient-assessed integration of healthcare services and treatment burden among patients with multimorbidity?

## Introduction

Attending multiple outpatient clinics in hospitals is common for patients with multimorbidity [1,2,3]. The number of chronic conditions increases with rising age [4,5], which further amplifies the outpatient activities [5,6]. Of the adult population in Denmark, 29% has multimorbidity [1]. One-fourth of these patients have several trajectories in outpatient clinics; 4% of these patients have two or more outpatient trajectories. This group accounts for more than one-third of all outpatient contacts [1]. In Denmark, the number of patients attending multiple outpatient clinics has nearly doubled over a decade [7].

Providing adequate and correct care poses challenges, particularly for those with complex multimorbidity [8,9,10]. There is a risk of fragmented care if the care services are delivered without integrating the care provided by different medical specialties [11,12,13]. Research has shown that many care providers do not consider comorbidities or do not sufficiently address diffuse symptoms or problems, and some medication lists contain medications that are inappropriate for many patients with multimorbidity [14]. However, research is sparse on hospital-based interventions aiming to integrate care though enhanced collaboration between different medical specialties and professions [3,15,16,17] and through alignment of multiple scheduled appointments across specialised outpatient clinics [3]. Consequently, interventions aiming to integrate different outpatient appointments are needed. In a former study, we investigated the feasibility of a multidisciplinary outpatient pathway (MOP) for patients with multimorbidity who attended several outpatient clinics [3]. The intervention, which was based on elements of the Chronic Care Model and the SELFIE framework [18,19], aimed to coordinate outpatient appointments through enhanced collaboration across medical specialties. It also aimed to promote integrated care, which is seen as holistic, patient-centred and coordinated care between healthcare professionals [18,20].

In the present study, we aim to investigate the effects of the MOP on healthcare utilisation and on the patient-perceived integration of healthcare services and treatment burden.

## Methods

### Setting

The Danish healthcare system is financed through taxation. Residents in Denmark are entitled to free healthcare, including healthcare services in general practice and at hospitals. Almost all Danish residents (98%) are registered with a specific general practice [21], which they must consult first for medical advice and referral to hospital outpatient care [22]. Silkeborg Regional Hospital is situated in the Central Denmark Region (one of five regions in Denmark). At the time of the intervention, this hospital had two medical wards and nine medical specialities providing outpatient services.

### Design

We performed a quasi-experimental study of patients with multimorbidity [2] who attended two or more outpatient medical clinics at Silkeborg Regional Hospital from 15 August 2018 to 11 March 2020. In this study, the effects of the MOP were compared between those who entered the MOP and those who did not enter. The STROBE checklist was applied for reporting [23].

### Study population

Eligible patients for inclusion were ≥18 years of age, had two or more chronic conditions (for at least six months) and had upcoming scheduled appointments in two or more outpatient clinics in the medical specialities of pulmonology, cardiology, nephrology, rheumatology and endocrinology at Silkeborg Regional Hospital. We excluded patients if their attendance in an outpatient clinic was related to other research projects, diagnostic purposes or procedures without an accompanying care appointment in another outpatient clinic.

### Allocation to the intervention

Pathway coordinators received monthly lists of upcoming medical outpatient appointments; these appointments were registered in the electronic health records and were scheduled no more than six weeks apart. Whenever alignment of outpatient appointments was feasible, patients were allocated to the intervention, as reported in our previous paper. In majority of cases, one appointment date was kept and the others were rescheduled around it [3]. In some cases, alignment was not possible for various reasons [3]. Listed patients who were not allocated to the intervention formed the comparison group.

### Intervention

**Figure 1:**
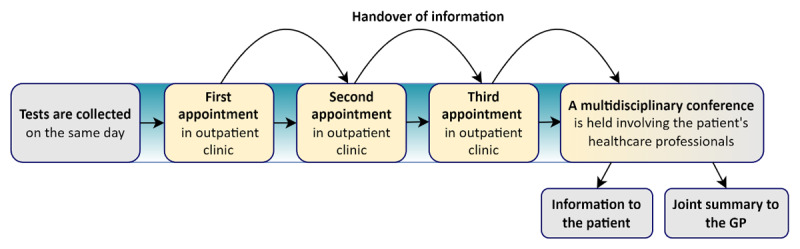
The Multidisciplinary Outpatient Pathway.

The MOP gathered scheduled medical appointments. As usual, outpatient appointments were attended individually, but they were scheduled to take place consecutively on the same morning. Laboratory testing was also scheduled on one date, mostly a few days before the hospital visit. After the first outpatient appointment, the patient would proceed to the following appointment. The order of appointments depended on how the coordination could be resolved. The delivering speciality wrote a summary with care-related information that was rapidly passed on to the receiving speciality. When all the patient’s visits to outpatient clinics had been concluded, the involved physicians and nurses from the different specialties attended a conference to agree on a joint care plan. The patient received verbal feedback, and the patient’s GP received a written summary, which stated that the intervention had taken place and described any modifications made to the individual care plans (Figure 1) [3].

### Baseline and follow-up

For the intervention group, baseline was defined as the date they received the intervention for the first time. For the comparison group, baseline was defined as the first date of entering the lists of pathway coordinators. All patients were followed for one year from baseline.

### Clinical and demographic information

Clinical and demographic information was obtained from the data warehouse in the Central Denmark Region. The data warehouse holds clinical information entered by clinicians into an electronic patient record, which is forwarded to the Danish National Patient Registry [24]. The included medical conditions were selected among the 39 conditions listed in the Danish Multimorbidity Index. Medical conditions were identified from the coding applied in accordance with the International Classification of Diseases, 10^th^ revision since 1993, whereas the Anatomical Therapeutic Chemical (ATC) classification system was used for specific conditions with repeated drug use at one year prior to baseline [25]. Information on age, gender, distance to hospital (shortest distance between hospital and home residence) and civil status (living with partner/living alone) was obtained from the Danish Civil Registration System [24].

### Care integration and treatment burden

Patient-reported information on chronic illness management, treatment burden, physical well-being and mental well-being was collected for intervention patients through questionnaires before and 2–4 days after entering the MOP. An additional questionnaire was sent to intervention patients at each time they experienced the MOP, and post-intervention replies were pooled.

Questionnaires were sent to the patient’s digital mailbox, which is used for electronic messages from all municipalities, regions and public authorities in Denmark, or were sent by letter with a self-addressed prepaid return envelope. Within two weeks, up to three reminders were sent to non-respondents; third reminders were sent by letter.

Chronic illness care was evaluated on the 20-item Patient Assessment of Chronic Illness Care (PACIC) questionnaire, which uses a 5-point response scale ranging from 1 (almost never) to 5 (almost always) [26]. Scores are aggregated into five care components that are central to the Chronic Care Model [27]: patient activation, delivery system design and decision support, goal setting and tailoring care, problem-solving and contextual counselling, and follow-up and coordination. Higher scores mean more frequent presence of the aspect of structured chronic care. Treatment burden was evaluated on the 10-item Multimorbidity Treatment Burden Questionnaire (MTBQ) with responses extending from 0 (not difficult/does not apply) to 4 (extremely difficult) [28]. A global score was calculated, ranging from 0 to 100, and four categories of treatment burden were generated by grouping the global scores into four main categories: no burden (score 0), low burden (<10), medium burden (10–22) and high burden (> = 22). Health-related quality of life was assessed though the 12-item Short Form Health Survey (SF-12) [29]. The SF-12 generates two separate mean scores; one for physical well-being and one for mental well-being. These scores range from 0 to 100; the higher the mean score, the higher the quality of life. Responses to the PACIC, MTBQ and SF-12 were included when ≥50% of items had been completed.

### Healthcare utilisation

We collected information from the data warehouse on provided hospital services during the one year before baseline and the one year after baseline; these data included information on inpatient and outpatient activities entered in the electronic patient record [24], blood samples registered in the laboratory information system [30] and GP contacts recorded in the regional health insurance data.

For healthcare services provided at Silkeborg Regional Hospital, we removed duplicates in the same speciality on the same day and counted the number of outpatient contacts and the use of different outpatient speciality clinics. To ascertain the number of hospital visits, the numbers of hospital visits for an outpatient appointment were accumulated for appointments on the same date. We counted the number of blood samples requested by the outpatient clinics, where blood samples refer to the actual times a patient’s blood sample was taken, not the number of requisitions (same-day blood sample requisitions were counted as one). We also counted the number of ended outpatient trajectories. The number of hospital admissions and the number of days of admission were calculated for all public hospitals in the Central Denmark Region. As data on admission and discharge contained overlapping periods, we identified the first date and the last date of each admission and then deducted the number of overlapping days to calculate admission time. We also found the number of GP daytime contacts, including emails and telephone consultations (codes 0101, 0201 and 0105), in the Central Denmark Region [31].

### Outcomes

The outcomes were patient-reported experience measures (PREMs), which were based on the PACIC questionnaire and the MTBQ completed by intervention patients (before versus after the intervention), together with the numbers of hospital visits, outpatient contacts, blood samples, terminated outpatient trajectories, hospital admissions, days of admission and GP contacts. All outcomes on healthcare provision were counted for the one year before and the one year after baseline to measure healthcare utilisation within and between groups.

### Ethical considerations

Inclusion was finalised before the COVID-19 pandemic affected outpatient activity in Denmark. No approval was required from the Committee on Health Research Ethics in the Central Denmark Region for this type of study. All participants were informed about the study from questionnaires, and upon returning a completed questionnaire they consented to the use of this information in research. Responses were securely stored in the Redcap web platform. The study was approved by the authority of the Central Denmark Region and listed as one of their studies (file no: 1-16-02-239-18), which enabled the collection of information from the data warehouse (file no: 1-45-70-48-21).

### Data analysis

Baseline characteristics of intervention group and comparison group were presented as counts with percentages, medians with interquartile intervals (IQI) or means with 95% confidence intervals (95% CI). Dichotomised variables were compared by using Fisher’s exact test. We used Student’s t-test for continuous data when normally distributed, otherwise Mann Whitney Wilcoxon test.

PREM outcomes were presented as counts and percentages, with a mean score and 95% CI for the PACIC questionnaire and a median score with IQI for the MTQB. To compare before-after PREM data on intervention patients, we used paired t-test or Wilcoxon’s signed rank test, depending on the distribution of data.

We modified a person’s at-risk time to account for death, emigration from the Central Denmark Region and hospital admission time (the outcome ‘days of admission’ accounted only for death and emigration). Then, incidence rates ratios were calculated (for one year before baseline and one year after) for the intervention group (IRR.i) and the comparison group (IRR.c). Using negative binomial regression with robust standard errors clustered at individual level, we analysed crude and adjusted incidence rate ratio ratios (IRRR = IRR.i/IRR.c) with 95% CIs for finding differences between periods and groups. This provided an output of modified effects on healthcare utilisation between groups. We adjusted for age, gender, specialities, and number of medical conditions, all as categorically grouped variables due to non-linearity. The fit of the analyses was examined by plotting the frequency of expected against observed values separately for the before and after periods by exposure groups. In sensitivity analyses, we used paired data to display the baseline characteristics of questionnaire non-respondents (Appendix 1). Analyses were performed with Stata, version 17.0.

## Results

### Study cohort

A total of 131 patients were assigned to the intervention group, and 524 patients formed the comparison group. The characteristics of the two groups were comparable at baseline, except that the intervention group had shorter distance to the hospital (7.5 kilometres (IQI, 2.8–17.9) vs. 8.2 kilometres (IQI, 3.3–21.7)), had more diagnoses (median 6 (IQI, 4–9) vs. 6 (IQI, 4–8)), were seen in more outpatient clinics (median 3 (IQI, 2–5) vs. 3 (IQI, 2–4)), had higher number of hospital visits (median 10 (IQI, 7–14) vs. 7 (IQI, 4–12)) and had higher number of outpatient contacts (median 10 (IQI, 7–14) vs. 8 (IQI, 5–13)) compared to the comparison group (Table 1).

**Table 1:** Baseline characteristics of intervention group and comparison group in the Multidisciplinary Outpatient Pathway (MOP) at Silkeborg Regional Hospital (SRH) from 15 August 2018 to 11 March 2020.


	INTERVENTION GROUP			COMPARISON GROUP			*p-VALUE*
	
	*N*	*%*	*MEDIAN (IQI)*	*N*	*%*	*MEDIAN (IQI)*	

Total	131			524			

Age			71 (61–76)			70 (61–77)	0.65

Female gender	55	42.0		226	43.1		0.84

Civil status							

Living with partner	43	32.8		208	39.7		

Living alone	88	67.2		316	60.3		0.16

Distance to hospital, km			7.5 (2.8–17.9)			8.2 (3.3–21.7)	0.04

Number of chronic conditions^a^			6 (4–9)			6 (4–8)	0.003

**Healthcare utilisation at one year prior to baseline** ^b^							

Hospital visits, SRH			10 (7–14)			7 (4–12)	<0.001

Hospital visits to the five selected outpatient clinics			5 (2–8)			4 (2–7)	0.11

Outpatient contacts, SRH			10 (7–14)			8 (5–13)	<0.001

Outpatient contacts to the five selected outpatient clinics			5 (2–8)			5 (2–8)	0.40

Outpatient clinics, SRH			3 (2–5)			3 (2–4)	<0.001

Outpatient clinics, other locations							

Cardiology	65	49.6		277	52.9		

Pulmonology	83	63.4		212	40.5		

Endocrinology	75	57.3		185	35.3		

Rheumatology	48	36.6		195	37.2		

Nephrology	27	20.6		74	14.1		<0.001

Contact to general practice, CDR			12 (6–25)			13 (6–21)	0.64

Hospital admissions, CDR							

Number of stays			1 (0–2)			1 (0–2)	0.28

Length of stays, days			3 (1–5)			2 (2–5)	0.49

**Treatment burden, MTQB** ^c^							

No burden, *score 0*	14	22.2		64	29.1		

Low burden, *score <10*	25	39.7		60	27.3		

Medium burden, *score 10–22*	21	33.3		60	27.3		

High burden, *score ≥22*	3	4.8		36	16.4		

Overall	63	48.1	*5* (2.5–12.5)	220	42.0	5 (0–15)	0.58

	***N***	***%***	***MEAN (95%CI)***	***N***	***%***	***MEAN (95%CI)***	***p-VALUE***

**Chronic illness care, PACIC** ^c^							

Patient activation	69	52.7	3.4 (3.1–3.7)	233	44.7	3.4 (3.2–3.5)	

Delivery system design/decision support	69	52.7	3.6 (3.3–3.8)	233	44.7	3.6 (3.5–3.7)	

Goal setting	69	52.7	2.7 (2.5–3.0)	233	44.7	2.6 (2.4–2.7)	

Problem-solving/Contextual counselling	68	51.9	3.1 (2.8–3.4)	230	43.9	2.8 (2.7–3.0)	

Follow-up/coordination	69	52.7	2.3 (2.0–2.5)	227	43.3	2.1 (1.9–2.2)	

Overall	69	52.7	2.9 (2.7–3.1)	334	44.7	2.8 (2.7–2.9)	0.24

**Well-being, SF-12** ^c^							

Physical health	64	48.9	32.2 (25.6–42.1)	218	41.5	37.8 (27.4–47.2)	0.87

Mental health	64	48.9	46.0 (36.8–56.5)	218	41.5	46.7 (39.1–54.0)	0.62


CDR: Central Denmark Region, IQI: Interquartile interval, km = kilometres, N: numbers, SRH: Silkeborg Regional Hospital. ^a^Out of 39 diagnoses from the Danish Multimorbidity Index. ^b^GP daytime consultations including email consultations and telephone consultations. ^c^Including persons responding ≥50%.

### Paired outcomes on PREMs for the intervention group

The overall mean PACIC score was 3.01 (95% CI, 2.77–3.26) at baseline and 2.9 (95% CI, 2.66–3.14) at follow-up with a response rate of 42% with no significant difference found (p-value = 0.15). For treatment burden, the overall median MTBQ score was 5 (IQI, 0–12) at baseline and 5 (IQI, 0–12.5) at follow-up, showing no significant difference between paired scores (p-value = 0.55) (Table 2). Respondents and non-respondents differed primarily on the characteristics of gender and civil status (Appendix 1).

**Table 2:** Questionnaire responses from intervention patients assessed before and after entering the Multidisciplinary Outpatient Pathway.


	TOTAL RESPONSE						PAIRED BEFORE-AFTER RESPONSES						
	
	*BASELINE*			*AFTER INTERVENTION*			*BASELINE*			*AFTER INTERVENTION*			*p-VALUE*
			
	*N, %*	*MEAN SCORE*	*95%CI*	*N, %*	*MEAN SCORE*	*95%CI*	*N, %*	*MEAN SCORE*	*95%CI*	*N, %*	*MEAN SCORE*	*95%CI*	

Total	131 (100)			131 (100)			131 (100)				131 (100)		

**Chronic illness care (PACIC)**													

Patient activation	69 (52.7)	3.4	(3.1–3.7)	73 (55.7)	3.4	(3.2–3.7)	55 (42.0)	3.4	(3.09–3.74)	55 (42.0)	3.4	(3.16–3.73)	

Delivery system design/decision support	69 (52.7)	3.6	(3.3–3.8)	73 (55.7)	3.6	(3.4–3.8)	55 (42.0)	3.7	(3.41–3.90)	55 (42.0)	3.6	(3.38–3.86)	

Goal setting	69 (52.7)	2.7	(2.5–3.0)	72 (55.7)	2.6	(2.4–2.8)	55 (42.0)	2.9	(2.60–3.15)	55 (42.0)	2.6	(2.31–2.87)	

Problem-solving/contextual counselling	68 (51.9)	3.1	(2.8–3.4)	72 (55.0)	3.0	(2.7–3.3)	55 (42.0)	3.2	(2.89–3.52)	55 (42.0)	3.1	(2.76–3.40)	

Follow-up/coordination	69 (52.7)	2.3	(2.0–2.5)	72 (55.0)	2.2	(2.0–2.5)	55 (42.0)	2.4	(2.08–2.69)	55 (42.0)	2.3	(2.01–2.56)	

Overall	69 (52.7)	2.9	(2.7–3.1)	73 (55.7)	2.9	(2.7–3.1)	55 (42.0)	3.0	(2.77–3.26)	55 (42.0)	2.9	(2.66–3.14)	0.15

	***N, %***	***MEDIAN SCORE***	***IQI***	***N, %***	***MEDIAN SCORE***	***IQI***	***N, %***	***MEDIAN SCORE***	***IQI***	***N, %***	***MEDIAN SCORE***	***IQI***	***p-VALUE***

**Treatment burden (MTQB)**													

No burden, *score 0*	14 (22.2)			22 (33.3)			12 (25.5)			16 (34.0)			

Low burden, *score <10*	25 (39.7)			20 (30.3)			18 (38.3)			15 (31.9)			

Medium burden, *score 10–22*	21 (33.3)			16 (24.2)			16 (34.0)			12 (25.5)			

High burden, *score ≥22*	3 (4.8)			8 (12.1)			1 (2.1)			4 (8.5)			

Overall	63 (48.1)	5	(2.5–12.5)	66 (50.4)	5	(0–12.5)	47 (36.0)	5	(0–12.5)	47 (36.0)	5	(0–12.5)	0.55


The numbers include persons responding to ≥50% to items. An average score was applied for intervention patients who entered the intervention more than one time. N: numbers, PACIC: Patient Assessment of Chronic Illness Care, MTBQ: Multimorbidity Treatment Burden Questionnaire, 95% CI: 95% confidence interval, IQI: interquartile interval.

### Comparing healthcare utilisation between groups

Most of the IRRs within-groups were under 1.0, meaning that hospital use was lower in the after period than in the before period. For hospital visits, a 19% reduction was seen between before and after period in the intervention group compared to the comparison group (IRRR: 0.81, 95% CI: 0.70–0.96). For blood samples, a 17% reduction was seen between before and after period in the intervention group compared to the comparison group (IRRR: 0.83, 95% CI: 0.73–0.96). The adjusted IRRRs showed no effect on outpatient contacts, ended outpatient trajectories, hospital admissions, days of admission and GP contacts (Table 3).

**Table 3:** Incidence rate ratios and incidence rate ratio ratios of healthcare utilisation (at one year after and one year before) for patients included in the Multidisciplinary Outpatient Pathway (MOP) intervention compared to patients not included from 15 August 2018 to 11 March 2020.


	CRUDE			ADJUSTED^d^		
	
	*IRR*	*IRRR*	*95%CI*	*IRRR*	*95%CI*	*p-VALUE*

SILKEBORG REGIONAL HOSPITAL						

**Hospital outpatient visits** ^a^						

Comparison group	0.90	0.79	(0.66–0.94)	0.81	(0.69–0.94)	

Intervention group	0.71					0.01

**Outpatient contacts**						

Comparison group	0.64	0.86	(0.71–1.03)	0.89	(0.76–1.04)	

Intervention group	0.55					0.13

**Blood samples** ^b^						

Comparison group	1.01	0.84	(0.72–0.96)	0.83	(0.73–0.96)	

Intervention group	0.85					0.01

**Terminated outpatient trajectories**						

Comparison group	1.39	0.90	(0.79–1.02)	0.91	(0.80–1.03)	

Intervention group	1.25					0.13

CENTRAL DENMARK REGION						

**Number of admissions**						

Comparison group	0.97	0.97	(0.91–1.04)	1.02	(0.97–1.08)	

Intervention group	0.96					0.43

**Days of admission**						

Comparison group	0.31	0.91	(0.46–1.79)	1.43	(0.69–2.96)	

Intervention group	0.36					0.39

**GP contacts** ^c^						

Comparison group	0.84	0.96	(0.83–1.09)	0.99	(0.86–1.13)	

Intervention group	0.80					0.83


Person time at risk was followed for one year from baseline until leaving the region, death, or end of follow-up. Time with admission was deducted person time at risk, except from the variable ‘Days of admission’. IQI: Interquartile interval, N: numbers, 95%CI: 95% confidence interval. ^a^Times going to Silkeborg Regional Hospital. ^b^Same-day blood samples requested from outpatient clinics. ^c^GP daytime consultations including email consultations and telephone consultations. ^d^Adjusted for age, gender, number of chronic conditions and number of specialties, as categorical variables.

## Discussion

This quasi-experimental intervention study investigated the effects of an intervention collating scheduled appointments in five specialised hospital-based outpatient clinics for patients with multimorbidity. We found that the number of hospital visits was reduced by 19%, and the number of blood samples was reduced by 17% in the intervention group. No effects were found on the patient-assessed quality of chronic care or treatment burden, the number of outpatient contacts, terminated outpatient trajectories, hospital admissions, days of admission or GP contacts.

The research is sparse on interventions for patients with multimorbidity, especially in the hospital setting [15,16,17,32]. To our knowledge, no prior studies have systematically aligned outpatient appointments. Some interventions have provided care coordination [33] or involved multidisciplinary teams in the patient care [34,35,36,37]. An example is the Clinic for Multimorbidity at Silkeborg Regional Hospital, where patients are referred by the GP. Similar to the MOP, a collaboration between medical specialties and healthcare professions provides a multidisciplinary conference and recommendations for the patient treatment [37].

Fragmented care for patients with multimorbidity has often been described in relation to hospital care. A study of patient records in Danish primary and secondary care found that comorbidities were not considered in two-thirds of patient cases, diffuse symptoms and problems were insufficiently addressed, and medication lists contained incorrect medications [14]. The same study found that barriers included relatively short consultation times, lack of care coordinators and lack of shared IT systems to provide an overview of the treatment [14]. There seems to be consensus that the management of multimorbidity requires a more patient-centered approach with greater integration and intensified coordination between the existing services [10,17,38,39,40,41]. This is also emphasised in models of care management, e.g. the Chronic Care Model and the SELFIE framework for integrated care for multimorbidity, which advocate for a holistic care approach that considers the patient’s health, capabilities and needs, environment, the systems surrounding the individual and contextual factors [18,19]. Our intervention embraces a collaborative approach, continuity and structural integration of outpatient care, although the approach to patient preferences and priorities was similar to usual care. We facilitated interaction and information-sharing across specialties to support decision-making, including shared responsibility for care decisions. Effective multimorbidity management should both improve the overall patient well-being and reduce the demand on healthcare services. However, while the MOP showed effects on hospital visits and blood samples, no improvements were seen on the variables related to the patients’ experience of provided care.

Many scheduled outpatient appointments will change over time (cancelled, re-scheduled or added). The nearer the monthly list was to the appointment date, the more stable these lists became, enabling effective care coordination. If the interval set for finding appointments was too wide, the pathway coordinators would have extensive work with going through the lists. As one appointment date was often kept, our intervention did not result in a complete reorganisation of all appointments, which otherwise may have negatively affected patients’ experience of treatment burden and care support. The order of consecutive outpatient appointments was determined by administrative factors and not by what would provide most clinical value. Deciding on a clinically meaningful sequence could complicate coordination and prove difficult for the coordinators to determine. Also, no limitations were set on diagnoses or the severity of medical conditions that would require collaboration. This influenced the composition of the study population and the number of appointments to be assessed and would add to the workload of assessing relevancy. Moreover, we excluded appointments for diagnostic purposes, therefore the study population’s actual number of outpatient visits might have been higher. This could influence responses about care support and treatment burden from intervention patients.

In a former process evaluation of the MOP, in the first year of the intervention, we describe how alignment was complicated by context. Subspecialisation and clinicians’ work schedules affected how many outpatient appointments could be aligned. In this prior study, we cover reach, fidelity and acceptability of the intervention [3]. Overall, intervention components were delivered as intended and seemed acceptable, although patient selection could be refined to enhance the relevancy of the intervention [3]. What was delivered, how much, for who and how, will influence how patients respond to experience measures related to intervention and the extent of healthcare utilisation.

Several strengths and limitations should be addressed. The novelty of the MOP adds to the limited number of hospital-based interventions for patients with multimorbidity. The paired observations of PREMs create a strong design, which reduces the risk of confounding and strengthens the statistical precision. Both the MTBQ and the PACIC questionnaire have been translated into Danish and have been found to provide valid and reliable measures of treatment burden and chronic illness care [26,42,43]. Healthcare utilisation is routinely and prospectively documented during clinical routines in hospitals and general practice, and the registers used contain information that is known to have high validity and completeness [22]. Moreover, we accounted for death, emigration from the region, admission time in the incidence rates of healthcare provision and for the variation of healthcare utilisation in different time periods.

An important limitation was the quasi-experimental design. Inclusion in the intervention group depended on the alignment of outpatient appointments, and different reasons may have determined the allocation to the intervention, as described in our former feasibility study [3]. To some extent, our design resembles that of a pragmatic trial although operational randomisation was not conducted. Blinding would not be feasible for this intervention and it adds to its strengths that few patients declined to participate [3]. Using monthly consecutive data lists allowed us to allocate study participants before their forthcoming outpatient appointment and to perform alignment. However, this method for patient selection this did not comply with a randomised design.

The response rates were low despite three reminders. However, we demonstrated that questionnaire respondents and non-respondents were largely comparable. Our response rate of 53% on the PACIC questionnaire at baseline was similar to the response rate of 54% reported in a Danish study on type 2 diabetes [42].

The IRRs were lower within groups after the intervention, but this can be explained. During the inclusion period, the registration method changed for outpatient care. This could have influenced the amount of registered outpatient activity. The Hawthorne effect may have caused the hospital staff in charge of bookings to increase their efforts to align outpatient appointments in both exposure groups. Any Hawthorne effect would have caused a non-differential effect by diminishing the difference for same-day outcomes, which could have made it more difficult to detect a change.

On the investigated outcomes, we found only statistically significant effects on reduced number of hospital visits and blood samples. It may be common sense that aligning events scheduled on separate days into one day will reduce the number of events. The patients in our study had outpatient appointments, which could not all be aligned [3]. Five specialties located at the same hospital were involved, but patients can attend outpatient appointments across hospitals or in the private sector. Thus, patients could have healthcare professionals involved in their care parallel to the intervention.

We interpret the effects with caution since the MOP did not reduce the number of outpatient contacts, end outpatient trajectories or improve on experience measures. Multimorbidity is a concept with diversity and embraces an assortment of patients with different needs for appointment alignment [3]. Even so, it is important to test these types of interventions and to prioritise the efforts in a healthcare system with limited resources. The results of this study may be generalisable to other countries with similar universal access to healthcare. Further research is needed on patients with multimorbidity and high outpatient healthcare demands and new models for coordinating hospital outpatient care.

## Conclusion

The MOP showed statistically significant effects in terms of reducing the number of hospital outpatient visits and the number of blood samples. No effects were found on patient-assessed chronic illness care, treatment burden, outpatient contacts, ended outpatient trajectories, hospital admissions, days of admissions or GP contacts.

## Data Accessibility Statement

The healthcare utilisation data that support the findings of this study are available from the Data warehouse of Central Denmark Region, but restrictions apply to the availability of these data, which were used under license for the current study, and so are not publicly available. PREM data are not publicly available because the consent to use these data are restricted to the current study.

## Additional File

The additional file for this article can be found as follows:

10.5334/ijic.7535.s1 Appendix 1. Characteristics of respondents and non-respondents.

## Figures and Tables

**Table:** 

ATC	Anatomical therapeutic chemical
CI	Confidence interval
GP	General practitioner
MOP	Multidisciplinary outpatient pathway
IRRs	Incidence rate ratios
IRRRs	Incidence rate ratio ratios
IQI	Interquartile interval
MTBQ	Multimorbidity Treatment Burden Questionnaire
PACIC	Patient Assessment of Chronic Illness Care (questionnaire)
PREMs	Patient-reported experience measures
SELFIE	Sustainable intEgrated chronic care modeLs for multi-morbidity: delivery, FInancing, and performancE
