# Supplementary material for: The Effect of Coordinating the Outpatient Treatment across Medical Specialities for Patients With Multimorbidity
Source: Int J Integr Care. 2024 Apr 9;24(2):4. doi: 10.5334/ijic.7535 (PMC11011960; doi:10.5334/ijic.7535)
Supplement: Appendix 1. — Characteristics of respondents and non-respondents. [file ijic-24-2-7535-s1.pdf]

Appendix 1. Characteristics of respondents and non-respondents [1]

	PACIC				MTBQ			
	Respondents (n=55, 42%)		***Non-respondents*** (n=76, 58%)		***Respondents*** (n=47, 36%)		***Non-respondents*** (n=84, 64%)	
	Mean	*95%CI*	*Mean*	*95%CI*	*Median*	*IQI*	*Median*	*IQI*
Age, median (IQI)	72	66-76	68.5	59-76	72	63-75	70	61-77
Female gender, N (%)	18	32.7	37	48.7	14	29.8	41	48.8
With partner, N (%)	41	74.6	47	61.8	11	23.4	52	61.9
Distance to hospital, km	7.1	2.8-18	7.6	3.0-17.8	7.8	3.3-18	7.4	3-17.8
Number of chronic conditions [a]	7	4-10	6	4-8	7	4-10	6	5-8
Outpatient contacts, SRH	11	7-14	10	7-14.5	10	6-14	10	7-15
Outpatient clinics, SRH	3	2-5	3	2-4	4	2-5	3	2-4
General Practice contacts, CDR [b]	10	5-25	13	6-24	10	5-25	29	14-51
Number of admissions, CDR	1	0-2	1	0-2	1	0-2	1	0-2
Days of admission, CDR	0	0-6	1	0-5	0	0-7	1	0-5

Respondents include those responding to ≥50% of items. Healthcare utilisation was estimated at one year prior to baseline. CDR: Central Denmark Region, IQI: interquartile interval, km= kilometres, MTBQ: Multimorbidity Treatment Burden Questionnaire, N: numbers, PACIC: Patient Assessment of Chronic Illness Care, SRH: Silkeborg Regional Hospital, 95%CI: 95% confidence interval. [a]Found among 39 medical conditions from the Danish Multimorbidity Index, [b]GP daytime consultations, including email consultations and telephone consultations.
